# Relative socioeconomic advantage and mood during advanced pregnancy in women in Vietnam

**DOI:** 10.1186/1752-4458-1-3

**Published:** 2007-08-09

**Authors:** Jane RW Fisher, Huong thu thi Tran, Tuan Tran

**Affiliations:** 1 Key Centre for Women's Health in Society, WHO Collaborating Centre in Women's Health School of Population Health, University of Melbourne, Melbourne, 3010, Australia; 2 Research and Training Centre for Community Development, Hai Ba Trung District, Hanoi, Vietnam

## Abstract

**Background:**

Mental health during pregnancy has not been investigated in Vietnam. Antenatal depression is an established risk factor for postpartum mood disturbance and two representative cohort studies have found rates of depression after childbirth in Vietnam two to three times higher than those in high income countries.

**Aim:**

The aim of this exploratory study was to investigate the prevalence and determinants of depression in a cohort of pregnant Vietnamese women. This was the subsidiary aim of an investigation of sexual beliefs and behaviours in pregnancy in Vietnam.

**Methods:**

Participants were recruited from antenatal clinics at Hanoi Obstetric Hospital. Inclusion criteria were to be in advanced pregnancy and appear well educated and confident and therefore potentially be willing to discuss sexual matters. Data were collected by individual structured interviews assessing socio-demographic characteristics, reproductive health, quality of intimate relationship and adequacy of support. Emotional wellbeing was assessed by the Vietnamese translation of the Edinburgh Depression Scale (EDS).

**Results:**

In total 61/74 (82%) of women approached to participate were recruited. The mean EDS score of 5.42 ± 3.8, was similar to that of community cohorts in high income countries and only one participant scored above 15. The cohort was relatively socioeconomically advantaged with high rates of post-secondary education, secure salaried employment, reproductive autonomy and ability to afford higher quality antenatal care. Most participants were able to confide in their husbands and their pregnancies were welcome. Worse mood was associated with insecure casual work, crowded living conditions and experiencing critical coercion in the marital relationship.

**Conclusion:**

There is an apparently low prevalence of symptoms of depression in relatively socioeconomically advantaged pregnant women in Vietnam.

## Background

Most of the research of the last four decades into the nature and determinants of perinatal mood disturbance in women has focused on the postpartum period and has been conducted in high income countries [1,2]. Recent investigations have established that depression is at least as common in pregnant women as in mothers of newborns and constitutes an independent risk for postpartum mood disorder [3]. In a comprehensive review Hendrick et al. [4] concluded that approximately 10% of pregnant women in the industrialized world experienced major depression and that the highest level of psychological symptoms occurred in the third trimester. There is growing evidence from low income countries that rates of postpartum depression are two to three times greater than in high income countries, but as yet there are only sparse data about the prevalence and determinants of depression during pregnancy in women living in low income countries.

A range of psychosocial factors has been associated with increased risk of depression in pregnancy including, adolescence, unwelcome conception, single marital status, unemployment and low income [5,6]. Early adverse experiences within family of origin, in particular recalled conflict and divorce appears to increase likelihood of depressive symptoms in pregnancy [7]. The quality of support available from a woman's own parents, in particular her mother; and from wider social support, including same-age peers is salient, but the relationship with her intimate partner is especially influential. Relationships that provide empathy, encouragement, opportunities to confide and practical support are protective against depression, but if women lack these relationships or if they are critical, intimidating and [5] hostile, the risk of poor mental health is increased [5,8,9].

There have been a small number of investigations of mood during pregnancy in low income countries. Fatoye et al. [10] found higher rates of depressive and anxious symptoms in pregnant women than in matched non-pregnant women in Nigeria. Depression was associated with having a polygamous partner, a prior termination of pregnancy and previous operative birth. In a small study of 33 low-income Brazilian women Da-Silva et al. [11] found that 12% were depressed in late pregnancy and that depression was associated with insufficient support from the partner and lower parity. Chandran et al. [12] interviewed a consecutive cohort of 359 women registered for antenatal care in a rural community in Tamil Nadu, India and found that 16.2% were depressed in the last trimester of pregnancy. Rahman et al. [13] established that 25% of pregnant women attending services in Kahuta, a rural community in Pakistan were depressed in the third trimester of pregnancy. In these latter studies risk was highest among the poorest women and those experiencing coincidental adverse life events and receiving insufficient assistance from others.

Vietnam is the second most densely populated country in South East Asia, with a population of approximately 83.5 million people, 74% of whom live in rural areas, most of these as subsistence farmers. In the last century Vietnam experienced three major wars, segregation from the non-communist world and more recently, the reforms of a free market economy and globalization. The Human Development Index is a composite score including average life expectancy, years of schooling and per capita income on which Vietnam is currently rated 112 out of 164 countries [14]. The average annual per capita income in 2003 was USD 2,490. There are disparities in relative poverty levels between those living in rural and urban settings, but it was estimated in 2004 that 20% of the population live in absolute poverty with a per capita income less than USD 1 per day [15].

There is emerging evidence that poor mental health is common in women in the postpartum year in Vietnam. Two detailed investigations using psychological autopsies to investigate maternal deaths (defined as those occurring during pregnancy or up to 42 days postpartum) in ten provinces have found that 8% to 16.9% are by suicide, which is exceptionally high by world standards [16,17]. Fisher et al. [18] found that 32.7% of 506 women attending immunisation clinics with their six week old babies scored in the clinical range of >12 on a translated and culturally verified version of the Edinburgh Postnatal Depression Scale and 19% expressed explicit ideas that they did not want to go on living. Tran Tuan et al. [19] found that 20% of the 2000 mothers of six to eighteen month old infants surveyed for the Vietnam arm of the Young Lives Project, an investigation of childhood poverty, met screening criteria for psychiatric clinical caseness on the locally validated WHO SRQ 20. Both of these studies surveyed representative samples of women who had recently given birth. Given that depression during pregnancy is a risk factor for depression after childbirth, these data indicate that antenatal depression might also be common in Vietnamese women. Neither published nor unpublished investigations of the prevalence and determinants of depression in pregnant women in Vietnam were found. The primary aim of this study was to investigate cultural beliefs about sexual behaviour in pregnant women in Vietnam, but the subsidiary aim reported here was to conduct a preliminary investigation of the prevalence and indicative determinants of symptoms of depression in the recruited cohort.

### Setting

The research was conducted in Hanoi, the capital of Vietnam which is located in the north of the country. Most women living in Vietnam give birth in medical settings at commune, district and provincial levels. Participants in this study were recruited from the National Obstetric Hospital, which is the main tertiary facility for obstetric care in the north of Vietnam. It provides a clinical service to women referred from the region with high risk pregnancies requiring specialist care. However, in addition, relatively socio-economically advantaged Hanoian women who constitute a minority of the population and may have low risk pregnancies, but seek high level obstetric care are able to pay for the Hospital's services.

### Sample and recruitment

This research was the subsidiary aim of a project that had been funded to investigate the sexual beliefs and behaviors of pregnant women in Vietnam as a postgraduate research report. Women observed to be in the second half of pregnancy and attending antenatal services at the National Obstetric Hospital in July and August 2004 were eligible to participate. Discussing sexual topics is highly sensitive and unfamiliar to Vietnamese women and only those who appeared to be confident, more advantaged and well-educated and therefore possibly more willing to discuss sexuality were approached. The sample size was limited by the constraints of a single student investigator being supported to undertake it in a limited time. The research was approved by the University of Melbourne Human Research Ethics Committee (HREC No. 040462); the Population Council who funded it and the Hanoi National Obstetric Hospital.

### Materials

The completion of self-report questionnaires about personal matters is an unfamiliar activity in Vietnam. Previous research experience had demonstrated that it was excessively time-consuming for informants and that individual structured interviews were preferred [18]. Data were therefore collected in this study in a four-part structured interview covering known determinants of perinatal mood disturbance in women. Sociodemographic details included: age; marital, educational and occupational status; and living circumstances. Reproductive health was assessed in terms of gravidity and parity, past history of spontaneous and induced abortions, current gestational age and whether the pregnancy was 'welcome' or convenient. Emotional support was measured via the self-assessed quality of a woman's relationships with her husband, mother, mother-in-law and peers. Relationship aspects included: sense of trust and ability to confide and potential for collaborative decision making. The assessment of women's sexual beliefs and sexual behaviors is described elsewhere [20]. The interview also included standardized psychometric instruments:

#### Edinburgh Depression Scale

The Edinburgh Postnatal Depression Scale (EPDS) is the most widely used screening measure for detection of major depression after childbirth with high sensitivity and specificity when using a clinical cut off score of 13 or more [21]. It is a ten item fixed choice measure which has been translated into multiple languages and validated for use in many cultural contexts [22]. It has also become widely used as a screening instrument for major depression in pregnancy when it is termed the Edinburgh Depression Scale (EDS) [5]. Each item is rated on a four-point scale (0 – 3), yielding a continuous score ranging from 0 to 30. In industrialized countries EDS scores of 13 or more are used to indicate clinically significant symptoms of postpartum mood disorder and a higher cut off score of 15 or more is recommended for detection of depression during pregnancy [23]. However, a clinical cut off of 13 or more has been used in most investigations of mood in pregnancy.

The EDS has been carefully translated into and back-translated from Vietnamese and checked for cultural accuracy for use in studies comparing Vietnamese born with locally born Australians [24,25]. Further modifications were required when it was used for the first time in Vietnam [18]. In order to clarify the meaning of colloquial English expressions which were not when translated literally comprehensible in Vietnam, Item 6: *Things have been getting on top of me *was altered to say *Do you feel that you have too many tasks to manage*? and item 10: *I have had thoughts of harming myself *was altered to say *Have you had thoughts that you do not want to live any more, and if so, how often*?. Fisher et al. [18] found that scores of 13 or more were significantly more common among women with classical symptoms of depression than in those without these symptoms.

#### Intimate Bonds Measure

The Intimate Bonds Measure (IBM) [26] is a 24 fixed-choice item self report questionnaire assessing quality of relationship with an intimate partner. It yields two subscales: 'Care' which assesses sensitivity, empathy, kindness and trust, and 'Control' which assesses critical, coercion, controlling behaviors and dominance. Each item is scored on a four point scale (0 – 3) to indicate whether the descriptor is experienced very often, moderately, somewhat or not at all. This instrument has not yet been used in Vietnam and therefore in order to assess its comprehensibility, acceptability and utility, a subset of seven items representing the subscales and thought to be most readily understood in the Vietnamese context were selected for use. They were translated into Vietnamese and after pilot testing the language was refined for cultural relevance by HT who is a Hanoi resident.

### Procedure

Flyers advertising the study were posted in antenatal clinics at the Hospital. Women who met eligibility criteria were approached and given a detailed verbal description of the project. Those who agreed to participate were given the plain language statement and consent form and invited to complete the interview in a private room at the clinic at a convenient time, usually while waiting for the antenatal check up. All recruitment and interviews were conducted at the Hospital antenatal clinics by HT who was experienced in social research about sensitive matters in Vietnam.

Prior to implementation the structured interview was pilot tested with four pregnant women attending the Hospital. As a result the order of administration of the sections was altered to: background information, recent pregnancy, relationships with husband and others, sexual behaviors and beliefs and then emotional wellbeing.

### Data analysis

Data were entered into a Statistical Package for the Social Sciences (SPSS) v12 spreadsheet. Separate sub-totals were calculated for scores on the groups of items drawn from the Care and Control subscales of the Intimate Bonds Measure. Three items (5, 14 and 16) from the Care subscale addressing feelings of being appreciated and needed, of being shown affection and experiencing sensitivity were summed to provide an Affection score. The potential range of scores was from 0 – 9, with higher scores indicating greater affection. Four items drawn from the Control subscale (9, 15, 20, 22,) assessed perception of coercion, control, criticism and efforts to change her, were summed to provide a Coercion score. The potential range of scores was from 0 – 12, with higher scores indicating greater coercion. Data were analysed by descriptive statistics and univariate measures of association including independent samples t-test, one-way ANOVA and, bivariate correlation and chi-square. All factors which were significantly associated with EDS scores were entered into a linear regression.

## Results

### Response rate and sample characteristics

In total 74 pregnant women were approached to participate and 61 agreed, a response rate of 82%. Reasons given for refusal to participate included discomfort about sharing private information and lack of time.

All participants were married, for an average of 3.5 ± 4.1 years and 67.2% (41/61) were nulliparous. They were aged 27.6 ± 4.6 years and their husbands were slightly older, 31.4 ± 5.3 years. Almost all (44, 72.1%) participants had completed a post-secondary qualification, which is around six times higher than the rate for parturient women in the Vietnamese population in general (13%, [19]; 12%, [18]. Reflecting this level of education, more than half (35, 57.4%) were in salaried employment in government or commercial sectors and the remainder were either currently unwaged and caring for older children or were self-employed in small businesses such as flower selling or tailoring. There was a highly significant association between education and employment. None of the 17 participants who lacked a post-secondary education were in salaried employment in the government or commercial sectors. However, 71% (31/44) of those with such qualifications were employed in this sector (χ^2^_1 _= 25.45, p < 0.0001). Most participants (55/61, 90.1%) felt that their employment or means of income generation was secure and would be available if desired after a period of maternity leave and 95.1% (58/61) that the household income was sufficient to meet their financial needs.

Traditionally Vietnamese people have lived in multigenerational households, but with increasing modernisation, more couples are living in nuclear households comprising only a married couple and their children [27]. Of these participants, 35/61 (57.4) were living in such a nuclear household; 23/61 (37.7%) in the woman's parents-in-laws' household and a small number of couples (4.9%) with the woman's own parents. Seven couples (11.5%) shared a bedroom with other family members and the remainder had a private bedroom.

### Reproductive health

Past reproductive losses were common in this cohort, 24/61 (40%) had experienced miscarriages and 28 (45.9%) a previous induced abortion. Overall almost half (29/61, 47.5%) had experienced either previous spontaneous or induced abortion, most of these (24/29, 82.7%) both. The pregnancy was reported to be very welcome by most participants (51/61, 85%) inconvenient, but now welcome by 8/61 (13%) and completely unwelcome by only 2/61 (3.3%). On average participants were in advanced pregnancy (29.2 weeks gestation ± 6.9 weeks) and all were in good health without identified pregnancy risk.

The questions in the structured interview were found to be understandable and meaningful and no-one refused to answer them.

### Quality of emotional relationship with husbands, families of origin and peers

For nearly half the participants (30/61, 49.1%) decisions about important aspects of their lives were made collaboratively between husband and wife, but for many women either their husband (17/61, 28.3%) or parents in law (8/61, 13.3%) made and imposed these decisions and only a few (6/61, 10%) women described themselves as the main decision maker. More collaboration was apparent in the decision to conceive the current pregnancy, for 42/61 (68.8%) women the husband and wife had decided jointly and seven (11.6%) women had made an independent decision, in the remainder either the husband (4/61, 6.7%) had decided or the pregnancy had been unintended (7/61, 11.7%).

The availability of trusted confiding relationships, especially with an intimate partner is found consistently to be protective of mental health. Most participants (49/61, 80.3%) felt able to confide anxieties and personal experiences in their husbands and many (28/61, 45.9%) also felt able to confide in their own mothers. However, the practice of confiding private experiences in a peer was not universal and only 22/61 (36.1%) indicated that they would share worries with a friend and 10/61 (16.1%) with a sister. Very few (9/61, 15%) confided in their mothers in law. On average women reported that they had three (± 0.9) confiding relationships and a third (32.8%, 20/61) that they had four or more. Only two women (3.3%) had no confiding relationships at all.

A woman's perception of her husband's daily attitudes and behaviours towards her during the pregnancy were assessed by items drawn from the Intimate Bonds Measure. The mean Affection score was 6.8 ± 1.56 with a range from 4–9. Most women perceived their husbands as affectionate and sensitive, only six women responded to an individual item as being *somewhat true *and none endorsed the *not true at all *option. There was more variation in the Coercion subscale on which the mean score was 2.3 ± 2.6 and range 0–11. Overall, 42.6% (26/61) reported that their husbands wanted to change them; 49.1% (30/61) that they were criticized in private; 47.8% (29/61) criticized over small matters and 19.6% (12/61) that they felt controlled in everything they did.

### Sexual relationship

Participants' sexual beliefs and experiences during pregnancy are described in detail elsewhere [20]. In brief, most (41/61, 67.2%) described a reduction from usual frequency of intercourse during pregnancy, but only 27.9% (17/61) felt that their current sexual relationship was not enjoyable and most (40/61, 65.6%) reported being generally satisfied with it. Non-penetrative forms of sexual pleasuring were rare, with no participants having experienced masturbation and only 6/61 (9.8%) oral genital sex.

### Edinburgh Depression Scale

There is debate about which EDS cut off score is most appropriate to indicate clinically significant symptoms in pregnant women in different populations and negligible evidence about this in low income settings, but various scores from > 9 to >15 have been utilised [23]. The EDS has not been validated for use in Vietnam and therefore locally established clinical cut off scores are not available. In this study 8/61 women (13.1%) scored above 9, including four who scored 12, but only one scored above 12 and also above 15. Three women explicitly acknowledged thoughts of wanting not to live anymore (Item 10), two of whom had these thoughts quite often. Although depressive symptoms were not widely reported, there were comparatively high scores on the EDS items which assess anxiety, with 62.3% (38/61) agreeing that they felt anxious and worried most or all of the time and 21.3% (12/61) that they felt scared or panicky without a specific reason. Green [28] has argued that the continuous EDS score is a valid indicator of variation in mood. The average EDS score in this study was 5.42 ± 3.8, range 0 to 18, which was significantly (p < 0.0001) lower than the mean score of 9.49 ± 6.32, range 0 to 26, found in the socioeconomically representative Vietnamese sample (Fisher et al [18] and similar to that reported by women in advanced pregnancy in England (Evans et al. [3]. Since comparisons could not be made between groups scoring in the clinical range of >15 with those scoring ≤ 14, further analysis to identify indicative social determinants of mood were made with continuous EDS scores.

### Associations with EDS scores

This was a homogeneous sample and there was no association between maternal age, length of marriage, level of education and EDS scores. However, lack of salaried work or a secure source of income and living in crowded conditions without a separate bedroom were associated with higher EDS scores indicating worse mood (see Table 1). The three women who reported ideas of not wanting to live anymore were all in non-salaried work.

**Table 1:** Factors associated with Edinburgh Depression Scale scores

Factors	N	Mean ± sd EDS score	t_59_	p
Employment status				
Salaried	31	4.42 ± 3.5	- 2.18	0.03
Trader, unskilled or casual work	30	6.47 ± 3.9		
Employment security				
Secure	55	5.07 ± 3.4	- 2.29	0.03
Not secure	6	8.67 ± 5.8		
Privacy				
Own bedroom	54	5.06 ± 3.3	- 2.19	0.03
Bedroom shared with family members	7	8.28 ± 5.8		
Pregnancy loss				
No past miscarriage	37	6.24 ± 4.1	2.16	0.04
Past miscarriage	24	4.16 ± 2.8		
Decision making about important matters				
Woman and her husband	36	4.75 ± 3.0	- 1.70	0.09
Husband and or in-laws	25	6.40 ± 4.5		
Decision to have this baby				
Woman and her husband	42	4.95 ± 3.4	- 1.75	0.09
Unplanned or decided by others	18	6.78 ± 4.26		
Criticism over small things				
Not at all	32	4.31 ± 3.3	- 2.52	0.01
Somewhat, moderately or very true	29	6.67± 3.91		
Controlled in everything I do				
Not at all	49	4.90 ± 3.39	- 2.28	0.03
Somewhat, moderately or very true	12	7.58 ± 4.64		

There were also no associations between gestational age, parity, and previous induced abortion. However, those who had a previous miscarriage had less mood disturbance than those who had not, perhaps interpretable as indicating relief that the safety of the current pregnancy was more assured. The small sample size precluded testing of significance, but the two participants who described the pregnancy as completely unwelcome had a mean EDS score that was almost double (11.00) that of the participants who regarded the pregnancy as welcome (5.56 ± 3.6).

In general this group was well supported emotionally and there was no relationship between mood and capacity to confide in particular people or the total number of confiding relationships. Women who lacked autonomy in making decisions, both about matters of life importance and about conceiving the current pregnancy had higher EDS scores than those who felt able to influence and collaborate in making these decisions, but these did not reach statistical significance. There were no relationships between current frequency of sexual intercourse, sexual satisfaction or sexual enjoyment and EDS scores.

There is consistent international evidence that the quality of relationship between a pregnant woman and her intimate partner is a determinant of mood [2]. In this cohort, there was no association between either the individual items, or the total Affection scale and EDS scores (Pearson correlation = -0.12, ns). However, higher scores on the Coercion subscale were associated with higher EDS scores (Pearson correlation = 0.36, p < 0.005). Two items contributed in particular to EDS scores: women who felt criticized over small things (t_59_= 2.52, p < 0.014) and who felt controlled by their partners (t_59_= 2.28, p < 0.03) had significantly lower mood than those who reported that they never had these experiences. It also appeared that psychosocial adversities accrued. Women who were not in salaried employment and those who lacked autonomy or participation in making important decisions had significantly higher Coercion scores than those who were securely employed or made collaborative decisions. While the differences were not statistically significant those whose pregnancies were unplanned or unwelcome and who felt that they lacked participation in the decision to have the pregnancy had higher Coercion scores than those whose pregnancies were welcome and chosen (see Table 2).

**Table 2:** Factors associated with Coercion Scale

Factors	N	Mean ± sd EDS score	t_59_	p
Employment status				
Salaried	31	1.61 ± 2.39	- 2.29	0.03
Trader, unskilled or casual work	30	3.10 ± 2.67		
Decision making about important matters				
Woman and her husband	36	1.72 ± 1.56	-2.30	0.03
Husband and or in-laws	25	3.24 ± 3.49		
Pregnancy				
Welcome	51	2.09 ± 2.41	-1.68	0.09
Inconvenient or unwelcome	10	3.60 ± 3.37		
Decision to have this baby				
Woman and her husband	42	1.97 ± 2.43	-1.70	0.09
Unplanned or decided by others	18	3.22 ± 2.96		

In order to identify the relationships between the factors for which there were significant univariate associations with lower mood, these were entered into a linear regression, with the EDS score as the dependent variable. Factors included income generating occupation, security of this occupation, availability of a private bedroom and the coercion scale. Together these factors accounted for 24% of the variance (r^2^) in the EDS. All the variables contributed significantly, but when the others are constant only the Coercion subscale remained significantly associated with the EDS scores (see Table 3).

**Table 3:** Linear regression of factors associated with EDS scores

Independent variable	Standardized Coefficient	t_59_	p	95% confidence Interval for β	
				
				Lower	Upper
Private bedroom 0 = Yes 1 = No	0.22	1.90	.06	- 0.15	5.43
Occupation 0 = Professional, salaried 1 = Trader, unskilled or casual	0.12	.95	.35	- 0.97	2.73
Employment security 0 = Secure 1 = Not secure	0.18	1.47	.15	- 0.84	5.46
Coercion subscale	0.27	2.21	.02	0.04	0.73

**Constant**		5.31	0.001	2.25	5.98

## Discussion

The limitations of this small exploratory study are that it had a convenience sample of modest size and used a psychometric measure, the Edinburgh Depression Scale that has been found comprehensible, but is yet to be validated in Vietnam. Paradoxically however, the study's most serious limitation emerged as a strength. The sample was significantly skewed with a six-fold over-representation of well-educated, securely employed women who despite healthy pregnancies chose to pay for antenatal care at a major specialist national obstetric hospital. Vietnam is among the poorest third of countries in the world and very few individuals are wealthy in world terms. However, since the Doi Moi economic reforms were introduced in 1986 there is a growing socioeconomic gradient in particular between the majority who live in rural areas and work in subsistence farming and the minority who live in urban areas with better access to education and salaried employment including in private companies [29]. Those who are professionally educated through post-secondary qualifications have much greater access to relatively well-paid, secure employment than those who have incomplete secondary or only primary education. In Vietnam women continue to have proportionately lower access to education than men, Haughton et al. [30] reported that 12.5% of women in the general Vietnamese population had completed post-secondary education, 8.5% had finished upper secondary school and 16.2% lower secondary school. The remaining two thirds of women had either completed primary education or had no formal education. Most government departments and many private companies are located in Hanoi which is the capital of Vietnam. This sample therefore represents a social minority who occupy the most advantaged position in the community [29]. Although this means that the findings cannot be generalised to the overall population of pregnant women in Vietnam, they have revealed an apparent social gradient in pregnancy psychological functioning that might not have been apparent in an investigation of an accurately representative sample.

Using the cut off score for detection of major depression during pregnancy established in Anglophone populations, the rate of clinically significant psychological symptoms appeared to be low in this sample. One interpretation is that a lower EDS cut off score is needed to identify clinically significant distress during pregnancy in Vietnam. Evidence in favour of this possibility is that the average scores of the three women who acknowledged the profound despair of not wanting to live anymore were not exceptionally high. However, an accurate cut off score cannot be established until the Scale has been formally validated in Vietnam against a standardised diagnostic psychiatric interview.

In this sample there were low rates of the risk factors for poor perinatal mental health that have been established in high income countries: unemployment; insufficiency or insecurity of income; single marital status; unwanted pregnancy or a lack of confiding relationships. There were high rates of the protective factors of salaried employment and income security, including positions available after maternity leave; intimate relationships characterised by affection and capacity to confide and reproductive choice. Living conditions were relatively comfortable with most having at least an individual bedroom and a substantial group living in a nuclear family arrangement. The alternative interpretation is therefore that there was actually a low rate of clinically significant mood disturbance in this group because they were protected by being in relative terms highly socially and economically advantaged.

In this cohort higher EDS scores indicating lower mood were associated with psychological and social adversity including experiencing criticism and coercion in the intimate partnership, overcrowded living conditions, low security of employment and unwelcome pregnancy. Although few women reported symptoms of sufficient severity to suggest clinically significant disturbance, these data indicate that these factors may contribute cumulatively to causing more severe mood disturbance and associated disability.

It is argued that relative rather than absolute disparities in socioeconomic status are associated with inequalities in health. Socioeconomic advantage confers benefits in addition to material wealth, in particular greater personal autonomy and control over salient aspects of life which have been shown to be protective against depression [31]. Chen et al. [32] found that in America, women's social position as reflected in their employment status, adequacy of earnings and autonomy about economic decision making and reproduction was significantly associated with symptoms of depression. On balance therefore these findings suggest that there is a social gradient in the perinatal mental health of women in Vietnam, a low income country, in which those who are relatively materially advantaged, have a high social position, maximal control over economic and reproductive circumstances and autonomy have much lower prevalence of symptoms of depression than women in the general local community. These findings provide further evidence that social circumstances are powerful determinants of perinatal mood status and challenge the assertion that it is attributable to hormonal or other neurochemical mechanisms.

In conclusion these data reveal substantial variation in prevalence of perinatal mood disturbance in a low income country related to women's social and economic circumstances. In settings in which few women give birth in specialist obstetric hospitals there is clear potential for selection bias and inaccurate conclusions in research that only recruits participants from these facilities rather than from the general community.

## Authors' contributions

JF supervised HT who conducted the investigation of women's sexual behaviours and beliefs during pregnancy as the research report required as a component of the Master of Women's Health. She designed the interview schedule about pregnancy mental health, analysed these data and wrote the paper. HT conducted the interviews, entered the data and contributed to analysis and interpretation. TT provided field supervision in Vietnam and contributed to analysis and interpretation of data.
